# Impacts of COVID-19 and partial lockdown on family functioning, intergenerational communication and associated psychosocial factors among young adults in Singapore

**DOI:** 10.1186/s12888-021-03599-z

**Published:** 2021-11-26

**Authors:** Wilson Wai San Tam, Sum Nok Poon, Rathi Mahendran, Ee Heok Kua, Xi Vivien Wu

**Affiliations:** 1 Alice Lee Centre for Nursing Studies, Yong Loo Lin School of Medicine, National University of Singapore, Level 2 MD11, 10 Medical Drive, Singapore, 117597 Singapore; 2 Department of Psychological Medicine, National University Hospital, Singapore, Singapore; 3 Department of Psychological Medicine, Yong Loo Lin School of Medicine, National University of Singapore, Singapore, Singapore

**Keywords:** COVID-19, Family functioning, Intergenerational communication, Psychological well-being, Young adults

## Abstract

**Background:**

The COVID-19 pandemic has changed our daily lives. Most of the working adults adopted the work-from-home arrangement while students shifted to home-based learning. Being confined together allows families to foster stronger bonds. On the other hand, the on-going pandemic could have negative impacts on family relationships. The COVID-19 outbreak is still on-going worldwide, understanding more about the changes in family functioning and its associated psychological impacts in a pandemic would allow the authorities to provide more targeted support to families.

**Objectives:**

This study aimed to examine the factors associated with family functioning among young adults in Singapore during the COVID-19 pandemic. Family functioning refers to the quality of interactions among family members, and consists of cohesion, flexibility and communication.

**Methods:**

A cross-sectional online survey was conducted (*N* = 390). The Family Adaptability and Cohesion Evaluation Scale Short Form (FACES-IV-SF) and Global Perceptions of Intergenerational Communication Scale (GPIC) were used to examine family functioning and intergeneration communication during the partial lockdown. Center for Epidemiologic Studies Depression Scale (CESD), Social Support Questionnaire–Brief (SSQ-B), Perceived Stress Scale 4 (PSS), UCLA Loneliness Scale, and Brief Resilient Coping Scale (BRCS) examined the psychosocial impact. Descriptive statistics, Pearson’s correlation coefficients, and regression model were employed in the analysis.

**Results:**

The FACES-IV-SF score for total circumplex ratio has a mean of 1.57(SD = 0.58), suggesting that participants generally perceived their families as functioning relatively well. The mean scores for CESD, PSS, Loneliness and BRCS were 12.4(6.2), 8.0(2.6), 5.7(1.9) and 12.6(3.1) respectively. The mean scores of the 4 domains of GPIC were 21.5(4.0) for Accommodation, 25.0(6.7) for Non-Accommodation, 17.2(3.3) for Respect-Obligation, and 18.9(4.8) for Avoidant.

**Conclusion:**

The results suggested that family functioning is significantly associated with intergenerational communication and satisfaction with social support in a pandemic. Participants with balanced levels of cohesion and flexibility in their families are more likely to be able to cope with the psychological impacts of the pandemic. The findings serve to inform intervention and preventive efforts to improve family functioning and reduce the risk of psychological distress in a pandemic.

## Highlights

• Family functioning is significantly associated with intergenerational communication

• Family functioning is significantly associated with satisfaction with social support

• Balanced levels of family cohesion and flexibility are more likely cope with psychological impacts of the pandemic

• Interventions are required to improve family functioning in a pandemic

• Preventive efforts are needed to reduce the psychological distress in a pandemic

## Background

The novel coronavirus (COVID-19) was first identified in Wuhan, China in December 2019 [1]. Since then, it has spread rapidly across the globe. On 11 March 2020, the World Health Organization (WHO) declared COVID-19 a global pandemic. As of 5 April 2021, the coronavirus has claimed over 2,867,019 lives across the world [2]. Major non-pharmaceutical interventions (NPIs) and lockdowns had a large effect on reducing transmissions across 11 European countries from February to May 2020 [3]. Italy is one of the worst-affected countries in Europe, NPIs such as the closure of schools and non-essential activities, and quarantine measures up to a generalised lockdown through the country were effective in flattening the curve and alleviated the pressure on the healthcare system [4, 5]. NPIs, including implementing lockdown measures, strict public health policy were effective in containing the spread of COVID-19 during the first half of 2020 in 131 countries [6]. In Singapore, the Disease Outbreak Response System Condition (DORSCON) alert level was raised from yellow to orange on 7 February 2020, signifying that the pandemic has moderate to high public health impact [7]. In response to the rising number of community cases, the authorities declared a partial lockdown (Circuit Breaker) of the country on 7 April 2020 [8]. During the two-month long “Circuit Breaker” period, Singaporeans were advised to stay at home as much as possible. Most of the working adults adopted the work-from-home arrangement while students shifted to home-based learning. All the non-essential services were closed, and social gatherings of people who are not from the same household were prohibited. The COVID-19 pandemic has thus changed the daily lives of Singaporeans.

Being confined together allows families more time to work on unresolved issues and support one another, which could translate to better and closer relationships. Consequently, some families have emerged with stronger bonds after the “Circuit Breaker” [9]. On the other hand, the on-going pandemic and the “Circuit Breaker” measures could have negative impacts on family relationships as well [10]. For instance, family conflicts could also arise more easily as individuals were confined to their homes with their family members for an extended period of time. In those cases, the high-pressure environment of confinement might complicate existing unresolved issues and also bring about new problems [11]. Moreover, other external stressors such as financial insecurity, worries about contracting COVID-19, increased burden of care on family members and potential job loss could also contribute to an increase in disagreements and conflicts within households [12, 13]. To date, the full consequences of this ongoing pandemic and the “Circuit Breaker” measures on family functioning have yet to be understood.

Family functioning refers to the quality of interactions among family members, as well as relationships, organization, expectations and support to each other [14]. The Olson Circumplex Model [15] conceptualises family functioning as containing three dimensions of family systems: cohesion, flexibility and communication. Family cohesion refers to the emotional bonding among family members. Flexibility is defined as the amount of changes in a family’s roles, rules and leadership, while communication refers to the exchange of information among family members. Balanced levels of cohesion and flexibility are associated with healthy family functioning. On the other hand, unbalanced levels of cohesion (i.e., disengaged or enmeshed) and flexibility (i.e., rigid or chaotic) are seen as problematic for family functioning in the long run [15].

Earlier research has found a strong link between intergenerational communication and family functioning [16, 17]. Intergenerational communication refers to the way verbal and non-verbal information is exchanged between two distinct generations (e.g., parent vs. child; grandparent vs. grandchild; baby boomers vs. millennials) [18]. Such communication is important within the family as it allows members to share their concerns and needs. It is also through communication that family members establish rules, provide support and resolve any conflict in the family [19]. Previous studies revealed that the pattern of speaking and interacting between the generations could affect the well-being of the family relationship [20–22]. Different family members have different communication styles. Early work showed that dysfunctional communication styles, which often cause resentment and misunderstanding, could lead to a breakdown in a relationship [23]. Thus, it would be interesting to examine how intergenerational communication is associated with family functioning in the context of a pandemic and partial lockdown.

Besides contributing to changes in family life, the COVID-19 pandemic and the associated restriction measures also have had a profound impact on the psychological well-being of individuals [24–27]. A recent study found that lockdown or social distancing measures could lead to feelings of loneliness in individuals due to the disruption of social life [28]. In addition, the uncertainty of the pandemic, and threats to health and financial security could also place individuals at risk for emotional distress such as anxiety and depression [29, 30]. Individuals’ negative emotions may spill over to their families, and such emotional spillover could potentially affect the quality of family interaction [31]. Likewise, a strain in family relationships could also contribute to emotional distress in individuals.

Social support and resilient coping are widely recognised protective factors against psychological distress [32, 33]. Prior studies found that social support and resilient coping were significantly associated with reduced psychological distress such as depression, anxiety and stress symptoms [34–36]. While spending more time at home during this pandemic, family members might not be the primary source of social support for some individuals. It is possible that this pandemic and the “Circuit Breaker” have cut off individuals’ typical outlets for coping with stressors and their usual channels of support. Nevertheless, family functioning is directly associated with psychological well-being of family members [37]. However, very few studies have investigated the relationship between family functioning and psychological well-being in the context of a pandemic.

As the COVID-19 pandemic and the related restriction measures have resulted in increased family time for most families, there is a need to expand our knowledge in relation to how this pandemic has impacted family functioning. The COVID-19 outbreak is still on-going worldwide, understanding more about the changes in family functioning and its associated psychological impacts in a pandemic would allow the authorities to provide more targeted support to families. It is yet to be known whether the COVID-19 pandemic and the resulting lockdown have contributed to stronger bonds or greater conflicts in families, and what are the associated psychological impacts. As such, the current study sought to examine the factors associated with family functioning among young adults in Singapore during COVID-19 pandemic. It is hypothesized that family functioning is associated with intergenerational communication, depressive symptoms, perceived stress, loneliness, social support and resilient coping.

## Methods

### Design and setting

A cross-sectional online survey was conducted to examine the perceived family functioning among young adults in Singapore. Eligible criteria were (i) full time university student, (ii) aged 18 or above, and (iii) family lived in Singapore.

### Sample size estimation

The Family Adaptability and Cohesion Evaluation Scale Short Form (FACES-IV-SF) included 8 domains, each with 3 questions, and the score of individual domain varies from 3 to 15. Assuming a normal distribution, the standard deviation (SD) of the score is approximately (15-3)/6 = 2. As the total number of fulltime students was about 30,000 [38], a sample size of 380 is required to achieve a margin of error of 0.2 (i.e. 10% of the SD) for the 95% confidence interval [39].

### Data collection

In September 2020, a recruitment message was posted in the Research Recruitment Board of LumiNUS (learning management system in the university), which could be accessed by all students. A brief description of the study was shown on the poster and the QR code as well as the URL of the e-survey were provided. Upon clicking the URL link or scanning the QR code, a participant information sheet and the electronic consent form were shown and the participant could proceed to the e-survey upon giving consent. The e-survey was created using the Qualtrics, a web-based survey solution, provided by the Information Technology Centre of the University [38]. Once the students clicked the ‘submit’ button, the data would be uploaded to the university server. It took approximately 10 to 15 min to complete the questionnaire. The e-survey was closed in October 2020 after reaching the desired sample size.

### Outcome measures

The questionnaire included seven instruments and demographic information. The FACES-IV-SF and Global Perceptions of Intergenerational Communication Scale were used to examine the family functioning and intergeneration communication during the circuit breaker. Center for Epidemiologic Studies Depression Scale, Social Support Questionnaire–Brief, Perceived Stress Scale 4, UCLA 3-Item Loneliness Scale, and Brief Resilient Coping Scale were used to examine the psychosocial impact.

#### Family adaptability and cohesion evaluation scale short form (FACES-IV-SF)

FACES-IV-SF was designed to assess family functioning based on Olson’s Circumplex Model (2011) [40]. FACES-IV-SF contained 24 items with the eight subscales measuring Balanced Cohesion, Balanced Flexibility, Disengagement, Enmeshment, Rigidity, Chaos, Communication and Satisfaction, respectively [41]. All the items included in the scale were measured on a 5-point Likert scale. In this study, ratio scores for cohesion and flexibility dimensions were calculated, using the following equations: 1) Cohesion Ratio Score = Balanced Cohesion/[(Disengaged + Enmeshed)/2]; 2) Flexibility Ratio Score = Balanced Flexibility/[(Rigid + Chaotic)/2]; 3) Total Ratio Score = (Cohesion Ratio Score + Flexibility Ratio Score)/2. Ratio scores for both cohesion and flexibility summarised the perceived level of family functioning within these dimensions. On the other hand, the total ratio score was utilised to assess the extent to which the family system is balanced or unbalanced. The higher the ratio score (above 1), the more balanced the family system, meaning that the family has healthier functioning. Conversely, the lower the ratio score (below 1), the more unbalanced the family system. The Cronbach’s alphas for the subscales ranged from 0.63 (Enmeshment) to 0.93 (Satisfaction) with a median of 0.81 [41].

#### Revised global perceptions of intergenerational communication scale (GPIC)

GPIC was developed by McCann (2003) to measure perceptions of intergenerational communicative experiences. The revised version was developed by Keaton and McCann [42] and used in Thailand and the US samples [43]. It consists of 25 items rated on a 5-point scale. The scale assesses perceptions of others’ communication on Accommodation and Non-Accommodation, and perceptions of one’s own communication on Respect-Obligation and Avoidant. The Cronbach’s alphas for Accommodation, Non-Accommodation, Respect-Obligation and Avoidant were respectively 0.79, 0.79, 0.67 and 0.79 [42].

#### Center for epidemiologic studies depression scale (CES-D-10)

CES-D-10 is a self-reported 10-item measure of depressive symptoms [44]. The frequency of each symptom during the past week is rated on a four-point scale. It has suggested cutoff scores of 8 and 10 in non-clinical samples [44]. Individuals with a score of 8 or more are at risk of depression. CES-D-10 was used in the Singapore population with good internal consistency [45, 46].

#### Social support questionnaire (SSQ)

SSQ measured the availability of social support, and level of satisfaction with social support using a 5-point scale [47]. The two items from SSQ yield separate scores for (a) perceived number of social supports (SSQ-N) and (b) satisfaction with available social support (SSQ-S), with higher scores reflecting higher perceived social support. SSQ-B was used in the local context with good reliability (α = 0.9) [48].

#### Perceived stress scale 4 (PSS-4)

PSS-4 is a four-item version of the Perceived Stress Scale [49], which measures the degree to which situations in an individual’s life were appraised as stressful, using a five-point scale. A PSS-4 score of 6 and more indicates a high level of stress [50]. The internal reliability of PSS-4 was shown to be good [51] and was 0.60 in this sample.

#### UCLA loneliness scale

The UCLA 3-Item Loneliness Scale is reliable for measuring loneliness, which scored on a 3-point scale [52]. The scores for the individual question are summed to give a total score ranging from 3 to 9, with scores 3 to 5 classified as “not lonely” and scores 6 to 9 as “lonely”. Excellent internal consistency was reported from previous study [53] and was 0.85 in this sample.

#### Brief resilient coping scale (BRCS)

BRCS is designed to measure stress coping tendencies, with 4 items rated on a 5-point scale [54]. The sum of the four items gives a total score ranging from 4 to 20, with scores of 4 to 13 indicating low resilient coping, 14 to 16 indicating medium resilient coping and 17 to 20 indicating high resilient coping [54]. Excellent internal reliability was reported in a sample of tertiary students [55] and was 0.80 in this sample.

### Data analysis

Descriptive statistics including mean, standard deviation, frequency and percentage, were used to summarize the demographic as well as the psychosocial variables. The internal consistency of the scale was evaluated by Cronbach’s alpha. Pearson’s correlation coefficients were computed between the eight subscales of FACES-IV-SF and the demographic/psychosocial variables. When variables had the *p*-value of the correlation coefficients < 0.1 with the FACES-IV-SF subscale, they would be entered into regression analysis to examine their effects on the 8 domains for the FACE-IV-SF. All the regression models were adjusted for age, gender, year of study, ethnicity and faculty. Backward selection method was used to remove insignificant variables in the regressions. Jamovi version 1.6.40 was used for the statistical data analysis (https://www.jamovi.org/) and statistically significance was set at 5%.

### Ethical consideration

All research activities involved in this study received approval from the Institutional Review Board of the National University of Singapore (Approval No: NUS-IRB-2020-214). The survey was anonymous and a SG$1 (US$0.75) would be sent electronically to the respondent as a token of appreciation given he or she provided the mobile number for online transfer.

## Results

A total of 435 students attempted the questionnaire and 390 completed it. The mean age of the students was 21.4 years (SD = 1.9) and 115 (29.5%) of them were male. 355 (91.0%) of them were Chinese, 359 (92.1%) stayed with parents and/or grandparents during the circuit breaker and 23 (5.9%) stayed in residential hall during that period. The average daily time that they spent with the family before and during the circuit breaker was 5.6 (4.5) and 12.7 (7.7) hours respectively, increased by 7.1 (6.3) hours on average (Table 1).

**Table 1:** Characteristics of the respondents (*N* = 390)

Characteristics	N(%) / Mean ± SD
Gender	
➢ Male	115 (29.5%)
➢ Female	274 (70.3%)
Age (years)	21.4 ± 1.9
Ethnicity	
➢ Chinese	355 (91.0%)
➢ Malay	9 (2.3%)
➢ Indian	19 (4.9%)
➢ Eurasian	1 (0.3%)
➢ Others	6 (1.5%)
Year of Study	
➢ 1	101 (25.9%)
➢ 2	114 (29.2%)
➢ 3	87 (22.3%)
➢ 4	78 (20.0%)
➢ 5 or above	10 (2.6%)
Faculty	
➢ Art and Social Sciences	117 (30.0%)
➢ Business	86 (22.1%)
➢ Computing	21 (5.4%)
➢ Design and Environment	20 (5.1%)
➢ Engineering	49 (12.6%)
➢ Medicine	21 (5.4%)
➢ Sciences	70 (17.9%)
➢ Others (Dentistry, Integrative Science and Engineering, and Law)	6 (1.6%)
Stay in Hostel during the Circuit Breaker	
➢ Yes	23 (5.9%)
➢ No	367 (91.4%)
Number of People Staying with during the Circuit Breaker	
0	14 (3.6%)
1	12 (3.1%)
2	64 (16.4%)
3	147 (37.7%)
4	94 (24.1%)
5 or above	59 (15.1%)
Living with	
➢ Parents only	62 (16.4%)
➢ Grandparents only	4 (1.1%)
➢ Parents and Grandparents only	10 (2.6%)
➢ Parents and Siblings only	218 (57.5%)
➢ Grandparents and Siblings only	2 (0.5%)
➢ Parents, Grandparents and Siblings only	26 (6.9%)
➢ Parents, Siblings and Others	26 (6.9%)
➢ Parents, Grandparents, Siblings and Others	10 (2.6%)
➢ Parents and Grandparents and Others	1 (0.3%)
➢ Others	31 (7.9%)
Average Time Spent with Family before Circuit Breaker (Hours)	5.6 ± 4.5
Average Time Spent with Family during Circuit Breaker (Hours)	12.7 ± 7.7
Difference of Time Spent with Family during & before Circuit Breaker (Hours)	7.1 ± 6.3

Table 2 showed the mean scores for the psychosocial variables. The mean scores for the 8 subscales of FACES-IV-SF ranged from 6.9 (Chaotic) to 10.8 (Balance Cohesion) with a median of 9.1. The FACES-IV-SF score for total circumplex ratio has a mean of 1.57 (SD = 0.58), suggesting that participants generally perceived their families as functioning relatively well. The mean scores for CESD, PSS, Loneliness and Resilience Coping were 12.4 (6.2), 8.0 (2.6), 5.7 (1.9) and 12.6 (3.1) respectively. 298 (76.6%) participants scored 8 or more for CES-D, and 245 (62.9%) students scored 10 or more for CES-D. Also, 323 (83.0%) participants scored 6 or more for PSS-4, while 221 (56.8%) participants score 6 or more for the UCLA loneliness scale. 235 (60.4%) participants scored 13 or below for BRCS indicating low resilient coping. The mean scores of the 4 domains of Rev-GPIC were 21.5 (4.0) for Accommodation, 25.0 (6.7) for Non-Accommodation, 17.2 (3.3) for Respect-Obligation, and 18.9 (4.8) for Avoidant.

**Table 2:** Summary of the psychological variables

	Mean ± SD	Cronbach’s alpha
FACE – Balance Cohesion	10.8 ± 2.4	0.78
FACE – Balance Flexibility	10.6 ± 1.9	0.49
FACE – Disengaged	8.1 ± 2.6	0.65
FACE – Enmeshed	7.1 ± 2.3	0.56
FACE – Rigid	7.9 ± 2.8	0.75
FACE – Chaotic	6.9 ± 2.6	0.76
FACE – Communication	10.5 ± 2.6	0.84
FACE – Satisfaction	10.1 ± 2.8	0.92
Cohesion Ratio Score	10.3 ± 3.5	n.a.
Flexibility Ratio Score	10.1 ± 2.1	n.a.
Total Circumplex Ratio	1.57 ± 0.58	n.a.
CESD (Public Domain)	12.4 ± 6.2	0.83
PSS (No need permission)	8.0 ± 2.6	0.59
Loneliness (No need)	5.7 ± 1.9	0.85
Resilient Coping (Obtained)	12.6 ± 3.1	0.80
GPIC Accommodation	21.5 ± 4.0	0.87
GPIC Non-Accommodation	10.0 ± 6.7	0.87
GPIC Respect	17.2 ± 3.3	0.79
GPIC Avoidant	18.9 ± 4.8	0.79
SSQ N	2.9 ± 0.9	n.a.
SSQ S	3.7 ± 0.8	n.a.

*CESD* Center for Epidemiological Studies-Depression, *PSS* Perceived Stress Scale, *ResCop* Brief Resilient Coping Scale, *GPIC ACC* Accommodation, *GPIC* Non-Accommodation, *GPIC RES* Respect, *GPIC ADV* Avoidant, *SSQ N* Perceived number of social supports, *SSQ S* Satisfaction with social support

Table 3 shows the Pearson’s correlation coefficients between the 3 ratio scores of FACES-IV-SF and other variables, significant correlations were bold. After adjusting for the effects of the other explanatory variables, circumplex ratio is positively associated with accommodation communication (β = 0.047, 95%CI: 0.035, 0.059) and satisfaction with social support (β = 0.084, 95%CI: 0.032, 0.136), but negatively associated with non-accommodation communication (β = − 0.026, 95%CI: − 0.019, − 0.034) and avoidant communication (β = − 0.024, 95%CI: − 0.034, − 0.014). It seems that both positive perceptions of intergenerational communication and satisfaction with social support are related to more adaptive family functioning, but negative perceptions of intergenerational communication and the lack of communication between family members are linked to poor family functioning.

**Table 3:** Correlation of the variables with the 3 ratio scores of FACE-VI-SF

	Age	TimeDiff	CESD	PSS	Lonely	ResCop	ACC	NACC	RES	ADV	SSQ N	SSQ S
Cohesion Ratio	−0.057 (0.262)	0.013 (0.809)	**−0.168 (< 0.001)**	**− 0.193 (< 0.001)**	**− 0.186 (< 0.001)**	**0.140 (0.006)**	**0.559 (< 0.001)**	**− 0.534 (< 0.001)**	0.046 (0.369)	**− 0.406 (< 0.001)**	**0.244 (< 0.001)**	**0.204 (< 0.001)**
Flexibility Ratio	0.010 (0.838)	**0.106 (0.044)**	**−0.279 (< 0.001)**	**− 0.231 (< 0.001)**	**− 0.193 (< 0.001)**	**0.182 (< 0.001)**	**0.406 (< 0.001)**	**− 0.462 (< 0.001)**	− 0.033 (0.513)	**−0.352 (< 0.001)**	**0.215 (< 0.001)**	**0.246 (< 0.001)**
Circumplex Ratio	−0.027 (0.591)	0.064 (0.223)	**−0.249 (< 0.001)**	**− 0.238 (< 0.001)**	**− 0.212 (< 0.001)**	**0.180 (< 0.001)**	**0.544 (< 0.001)**	**−0.561 (< 0.001)**	0.008 (0.870)	**− 0.426 (< 0.001)**	**0.258 (< 0.001)**	**0.252 (< 0.001)**

*FACE BC* Balance Cohesion, *FACE BF* Balance Flexibility, *FACE DE* Disengaged, *FACE EM* Enmeshed, *FACE RD* Rigid, *FACE CH* Chaotic, *FACE CO* Communication, *FACE SA* Satisfaction, *FACE CDS* Cohesion Dimension Score, *FACE FDS*, *TimeDiff* Time difference spent with family before and during the circuit breaker, *CESD* Center for Epidemiological Studies-Depression, *PSS* Perceived Stress Scale, *ResCop* Brief Resilient Coping Scale, *GPIC ACC* Accommodation, *GPIC* Non-Accommodation, *GPIC RES* Respect, *GPIC ADV* Avoidant

Specifically, cohesion ratio is positively related to accommodation communication (β = 0.064, 95%CI: 0.050, 0.078), but negatively related to non-accommodation communication (β = − 0.027, 95%CI: − 0.017, − 0.036) and avoidant communication (β = − 0.027, 95%CI: − 0.039, − 0.015). On the other hand, flexibility ratio is positively associated with difference in time spent with family before and during the “Circuit Breaker” (β = 0.013, 95%CI: 0.005, 0.021), accommodation communication (β = 0.034, 95%CI: 0.019, 0.049) and satisfaction with social support (β = 0.090, 95%CI: 0.023, 0.157), but negatively associated with depressive symptoms (β = − 0.013, 95%CI: − 0.022, − 0.004), non-accommodation communication (β = − 0.026, 95%CI: − 0.016, − 0.035), avoidant communication (β = − 0.016, 95%CI: − 0.028, − 0.003). Contrary to hypothesis, family functioning is not significantly associated with perceived stress, resilient coping, respect/ obligation communication and number of social support (Table 4).

**Table 4:** Linear regression analysis for the 8 domains of FACE-IV-SF. Backward selection method was used to identify the final models

	TimeDiff	CESD	ACC	NACC	ADV	SSQ S
Cohesion Ratio			0.064 (0.050, 0.078) < 0.001	−0.027 (− 0.017, − 0.036) < 0.001	−0.027 (− 0.039, − 0.015) < 0.001	
Flexibility Ratio	0.013 (0.005, 0.021) 0.002	− 0.013 (− 0.022, − 0.004) 0.005	0.034 (0.019, 0.049) < 0.001	−0.026 (− 0.016, − 0.035) < 0.001	−0.016 (− 0.028, − 0.003) 0.015	0.090 (0.023, 0.157) 0.008
Circumplex Ratio			0.047 (0.035, 0.059) < 0.001	−0.026 (− 0.019, − 0.034) < 0.001	−0.024 (− 0.034, − 0.014) < 0.001	0.084 (0.032, 0.136) 0.001

Adjusted for age, gender, ethnicity, year of study, and faculty

*FACE BC* Balance Cohesion, *FACE BF* Balance Flexibility, *FACE DE* Disengaged, *FACE EM* Enmeshed, *FACE RD* Rigid, *FACE CH* Chaotic, *FACE CO* Communication, *FACE SA* Satisfaction, *TimeDiff* Time difference spent with family before and during the circuit breaker, *CESD* Center for Epidemiological Studies-Depression, *PSS* Perceived Stress Scale, *ResCop* Brief Resilient Coping Scale, *GPIC ACC* Accommodation, *GPIC* Non-Accommodation, *GPIC RES* Respect, *GPIC ADV* Avoidant

## Discussion

The present study examined the associations between young adults’ perceptions of family functioning, intergenerational communication, and their reported depressive symptoms, perceived stress, loneliness, resilient coping and social support during the COVID-19 pandemic “Circuit Breaker” period in Singapore. As predicted, intergenerational communication is significantly associated with family functioning. Specifically, higher levels of accommodation communication are linked to greater family cohesion and flexibility. Conversely, higher levels of non-accommodation communication and avoidant communication are found to be related to lower family cohesion and flexibility. These results are in accordance with those from previous studies [20–22]. Families with better intergenerational communication tend to feel closer, more flexible in resolving conflicts and more satisfied with one another, as compared to families with less effective intergenerational communication [56].

On the other hand, these findings could also be interpreted as functional families are more likely to promote more adaptive intergenerational communication, while dysfunctional families tend to maintain negative communication patterns [15]. Moreover, it was found that family functioning is not related to respect/obligation communication. This suggests that speaking respectfully to the more senior family members might have little influence on family cohesion and flexibility. In addition, research by Jackson and colleagues [16] found a positive association between problematic intergenerational communication and an escalation of family conflict. Poor communication in the family systems is likely to cause misunderstanding and arguments, and consequently, worsen relationships among family members. Hence, it is possible that adaptive intergenerational communication may strengthen family functioning, which could serve as a protective factor against psychological distress during the COVID-19 pandemic.

The current findings suggest that the COVID-19 pandemic and the related restriction measures seem to have immediate negative impacts on the psychological well-being of university students. The results showed that about three-quarters of the participants are at risk of depression. Moreover, an alarming 83.0% of the participants reported experiencing high levels of stress. 56.8% of the participants scored 6 or higher on the UCLA loneliness scale, which shows that more than half of the participants felt lonely during the pandemic. Notably, these results are in line with findings from recent studies that investigated the psychological impacts of the COVID-19 pandemic on university students [57–60]. Some of the pandemic-related impacts such as disruption in daily routines, loss of social contact, financial hardship in the family, fear of death, uncertainty of the future could have contributed to psychological distress in students. Moreover, students may have older grandparents in the household who are at high risk of depression, post-traumatic stress disorder in the post-COVID-19 pandemic [5, 61]. Psychosocial support, like peer support, sharing of experiences, could promote resilience and coping strategies [5]. In addition, spiritual beliefs have been associated with higher level of hopefulness and lower level of fear, worry and sadness during the COVID-19 pandemic [62]. Spirituality is a recognised source of comfort, support and meaning for the individual, hence, spiritual resources could be an effective way of resilience to manage and cope with stressful events due to the pandemic [4].

Furthermore, the results revealed that lower family flexibility is associated with greater depressive symptoms. Families lower in flexibility are more likely to have higher levels of conflict, and this could increase the risk for depressive symptoms in individuals [63, 64]. On the other hand, more flexible families tend to communicate more openly, provide greater support and have better problem-solving ability, which might help to buffer against depressive symptoms [65, 66]. Interestingly, greater family flexibility is associated with greater differences in time spent with family before and during the “Circuit Breaker”. It could be that the increase in time spent with family during the “Circuit Breaker” created more opportunities to facilitate changes in leadership, roles and rules in the family.

In addition, the results showed that satisfaction with social support is positively related to family functioning. In particular, higher satisfaction with social support is associated with better family flexibility and more balanced family system. These associations are in line with findings from previous studies that examined families coping with stressful situations [67, 68]. One possible explanation for the observed associations is that individuals from more functional families tend to have stronger social support networks [69]. Individuals with greater satisfaction on social support tend to be better able to maintain a functional family. Furthermore, past research revealed that the quality of social support is associated with lower psychological distress [32, 34, 36]. Hence, it seems that social support may serve as a protective factor against the negative consequences of stressors such as family conflict or job losses due to the pandemic.

In summary, the results suggested that family functioning is significantly associated with intergenerational communication and satisfaction with social support in the context of a pandemic. Additionally, family flexibility is also associated with differences in time spent with family before and during the “Circuit Breaker” and depressive symptoms. It seems that young adults with balanced levels of cohesion and flexibility in their families are more likely to be able to cope with the psychological impacts of the pandemic. Nevertheless, it is important to note that there might be individual differences in responses to family processes [70]. Moreover, not everyone is affected by the pandemic and partial lockdown in the same manner. It is possible that other individual attributes, such as emotional reactivity are influencing these relations.

### Limitations and future directions

The nature of the COVID-19 pandemic is dynamic and ever-changing, findings from this study need to be interpreted with caution. The long-term consequences of this ongoing pandemic on families and individuals have yet to be investigated. Hence, future studies could employ a longitudinal design to explore the long-term impacts of the pandemic on family functioning and young adults’ psychological well-being. Moreover, as the data for this study were collected solely from university students in Singapore, it is unclear whether the present findings can be generalised to other population. Hence, future studies could extend the current work by recruiting a larger sample with greater demographic diversity. In addition, the cross-sectional nature of this study precludes any causal interpretation of relationships among the variables of interest. It is possible that many of the links between the variables examined in the study are bi-directional in nature. It would be meaningful to investigate the underlying directions of causality between the variables.

### Implications

Despite these limitations, this study provides evidence for the links between family functioning, intergenerational communication and psychological well-being during a global pandemic. This has notable implications for intervention. Considering the family functioning is tied to psychological well-being, it is important to keep family and psychological services easily accessible if national lockdown is required in the future. Lockdown measures are effective in curbing COVID-19 related infections and deaths and overburden on the healthcare system, but these measures are unsustainable for a long time due to economic and social issues [71]. Stakeholders could improve on the delivery of family counselling and mental health services to better support individuals remotely in a pandemic [61]. For instance, professional support could be made more accessible to families that struggle to resolve conflicts due to communication breakdown. Online training and self-help resources could help families to identify the existing maladaptive communication styles and adopt effective communication techniques [61]. Communicating in an empathic and sensitive manner would contribute to more positive interactions with family members, which in turn could improve the quality of family life and psychological well-being of individuals. Spiritual support could also provide resources for coping with the negative consequences of the COVID-19 pandemic [4].

## Conclusion

The Covid-19 pandemic and lockdown have impacted the daily lives of individuals and families in significant ways. The present study contributes to the literature by exploring the impacts of COVID-19 and the related restriction measures on family functioning and its associated factors among young adults. The current findings serve to inform intervention and preventive efforts aimed at improving family functioning and reducing the risk of psychological distress in the context of a pandemic.

## Data Availability

The datasets analysed during the current study are not publicly available due to limitations of ethical approval involving the participant data and anonymity but are available from the corresponding author on reasonable request.
